# Functional Outcomes of Cementless Total Hip Arthroplasty in Avascular Necrosis of the Hip: A Prospective Study

**DOI:** 10.7759/cureus.10136

**Published:** 2020-08-30

**Authors:** Sundas Karimi, Suresh Kumar, Faheem Ahmed, Awais Khalid, Umar Farooque, Fnu Shahzeen, Muhammad Ayoob Memon, Tooba Hussain, Maleeha Ali Basham, Naresh Kumar, Farah Yasmin, Syed Adeel Hassan

**Affiliations:** 1 General Surgery, Combined Military Hospital, Karachi, PAK; 2 Orthopaedics, Jinnah Postgraduate Medical Center, Karachi, PAK; 3 Orthopaedic Surgery, Trauma Centre Civil Hospital Karachi, Karachi, PAK; 4 Orthopaedics, King Edward Medical University, Lahore, PAK; 5 Neurology, Dow University of Health Sciences, Karachi, PAK; 6 Internal Medicine, Jinnah Sindh Medical University, Karachi, PAK; 7 Medicine, Dow University of Health Sciences, Karachi, PAK; 8 Internal Medicine, Dow University of Health Sciences, Karachi, PAK; 9 Cardiology, Dow University of Health Sciences, Karachi, PAK

**Keywords:** avascular necrosis, cementless total hip arthroplasty, functional outcomes, hip joint, harris hip score, modified harris hip score, humans

## Abstract

Introduction

Avascular necrosis occurs due to impaired blood supply to the bone. It can be caused by fractures, dislocations, chronic steroid use, chronic alcohol use, coagulopathy, congenital source, and many other factors. It mostly affects the femoral head (hip joint). Its management can be conservative or invasive. Total hip arthroplasty is the treatment of choice for third and fourth stage avascular necrosis that can be cemented or uncemented. The purpose of this study is to access the functional outcomes of cementless total hip arthroplasty in patients with avascular necrosis of the hip.

Materials and methods

This prospective study was conducted at a major metropolitan hospital in Karachi, Pakistan over a period of six months. A total of 30 patients of age <60 years, either gender, and a confirmed diagnosis of avascular necrosis of hip with no other associated hip pathologies were included in this study. Demographic features, comorbidities, level of activity, range of movement before the development of avascular necrosis, Charnley's class, and laterality were noted. Cementless press-fit extensively porous-coated acetabular cup with or without cancellous screws and cementless press-fit extensively hydroxyapatite coated femoral stem were used through modified Gibson's posterior approach. The patients were checked for early and late complications, the position of acetabular and femoral components by radiography, and overall performance by Harris Hip Score (HHS) and modified HHS over a period of 12 months. All statistical analyses were performed using Statistical Package for Social Sciences (SPSS) version 19.0 (IBM Corp, Armonk, NY).

Results

The mean age was 43.9±6.7 years with 21 (70%) patients ranging from 40 to 60 years of age. There were 22 (73%) male and 8 (27%) female patients. Nine (30%) patients had diabetes mellitus, eight (27%) had hypertension, two (7%) had other comorbidities, and eleven (37%) had no comorbidities. A total of 11 (37%) patients were highly active, 18 (60%) were moderately active, and 1 (3%) was non-active before developing avascular necrosis. There were 4 (13%) patients in Charnley's class I, 15 (50%) in Charnley's class II, and 11 (37%) in Charnley's class III. Fifteen (50%) patients were operated on the left side, seven (23%) on the right side, and eight (27%) bilaterally. No significant early or late complications were noted. Acetabular component was found to be anteverted in 22 (73%), retroverted in zero (0%), neutral in 8 (27%), <35^o^ inclined in 0 (0%), 35^o^-50^o^ inclined in 23 (77%), and >50^o^ inclined in 7 (23%) patients, while femoral component was found neutral in 28 (93%), valgus in 2 (7%), and varus in zero (0%) patients on radiography at follow-up. On functional assessment, the HHS was 100% in 27 (90%) patients, 96% in 2 (7%) patients, and 83% in 1 (3%) patient with an average of 99.2%, while 29 (97%) patients had excellent and only 1 (3%) patient had a good outcome on modified HHS.

Conclusions

Cementless total hip arthroplasty, performed in patients <60 years of age and avascular necrosis of the hip with no other associated hip pathologies, has excellent functional outcomes with no pain, limping, physical deformity, difficulty in walking, difficulty in climbing stairs, difficulty using public transport, difficulty in sitting, or difficulty in wearing shoes and socks. They usually attain normal limb length and range of movement.

## Introduction

The hip can be classified as the largest joint in the body. Its components include an acetabulum of the pelvic bone and a ball of the femoral head. The bony surfaces are protected with articular cartilage and smooth tissue that cushions the end of the bones and aids mobility in the joint [1].

Pathologies such as osteoarthritis, rheumatoid arthritis, post-traumatic arthritis, and avascular necrosis can profoundly affect the joint and become a source of pain and disability. Moreover, certain pathologies like avascular necrosis can result in the collapse of the femoral head in addition to secondary osteoarthritis. The phenomenon leading to avascular necrosis comprises impaired blood flow to the bone leading to bone cell death. Avascular necrosis commonly involves hip joint (femoral head) but may also occur in, shoulder, knee, and ankle joints [2]. Etiologies include fractures, dislocations, chronic steroid use, chronic alcohol use, coagulopathy, congenital source, and many others.

The management of avascular necrosis of the femoral head ranges from conservative to invasive. Conservative treatments, such as reduced weight bearing, physical therapy, discontinuation of steroid therapy, and anti-inflammatory medications, are recommended in the initial stages. Several pharmacological agents such as bisphosphonates and statins have shown clinical benefits in the early-stage of avascular necrosis [3,4]. Operative treatment can be classified into two categories: the first category includes procedures that preserve femoral head (core decompression, osteotomy, non-vascularized and vascularized bone grafting, and autologous bone grating), whereas the second category includes those procedures that partially or completely replace the femoral head (hemiarthroplasty, and total hip arthroplasty). However, total hip arthroplasty is the preferred treatment of choice for stage III and IV avascular necrosis that can be cemented or cementless [5].

This prospective study is aimed at evaluating the functional outcomes of cementless total hip arthroplasty in patients with avascular necrosis of the hip.

## Materials and methods

Study design

This prospective study was conducted at the Jinnah Postgraduate Medical Center Karachi, Pakistan from January 1, 2019 to June 30, 2019 over a period of six months. All patients aged <60 years of either gender (i.e male or female) with a confirmed diagnosis of avascular necrosis of the hip were included in this study. All patients aged >60 years and those with any other associated hip pathologies were excluded from this study.

Data collection

A total of 30 patients met the inclusion/exclusion criteria and were included in this study. All patients were informed about the purpose and aim of the study following which both verbal and written informed consents were taken. The demographic features (age, gender), comorbidities, level of activity, range of movement before the development of avascular necrosis, Charnley's class, and laterality were noted. All patients were operated by using modified Gibson's posterior approach. The cementless press-fit extensively porous-coated acetabular cup with or without cancellous screws and cementless press-fit extensively hydroxyapatite coated femoral stem were used. There were no significant events intraoperatively. Prophylactic antibodies were used from the day of surgery for up to 5-14 days. Patients were kept partial weight-bearing for four weeks and were mobilized progressively full weight-bearing thereafter. The patients were followed for 12 months for early and late complications, the position of acetabular and femoral components by radiography, and overall performance by Harris Hip Score (HHS) and modified HHS.

Data analysis

Data were entered and analyzed using Statistical Package for Social Sciences (SPSS) version 19.0 (IBM Corp, Armonk, NY). Continuous data were presented as mean and standard deviation, whereas categorical data were presented as frequencies and percentages.

## Results

The mean age of the patients was 43.9 years with a standard deviation of 6.7 years (Table 1).

**Table 1:** Analysis of age

Age (years)	Minimum	Maximum	Mean	Standard deviation
	20	60	43.9	6.7

**Figure 1:**
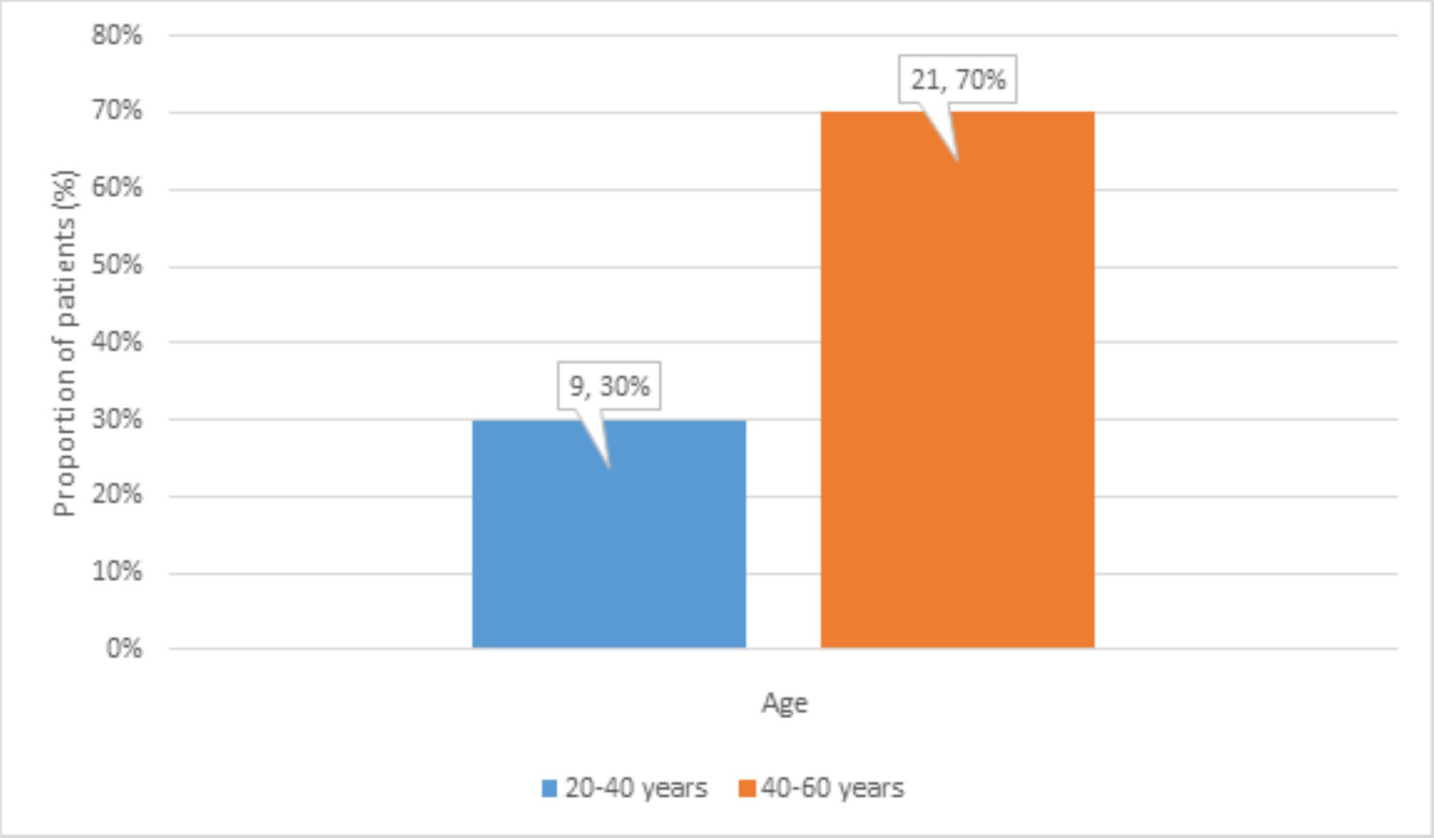
Distribution of age

There were 9 (30%) patients aged 20-40 years and 21 (70%) patients aged 40-60 years (Figure 1).

**Figure 2:**
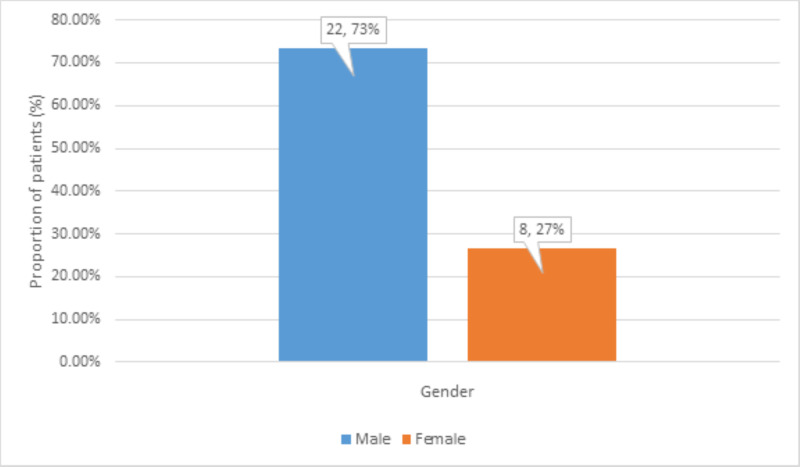
Distribution of gender

There were 22 (73%) male and 8 (27%) female patients (Figure 2).

**Figure 3:**
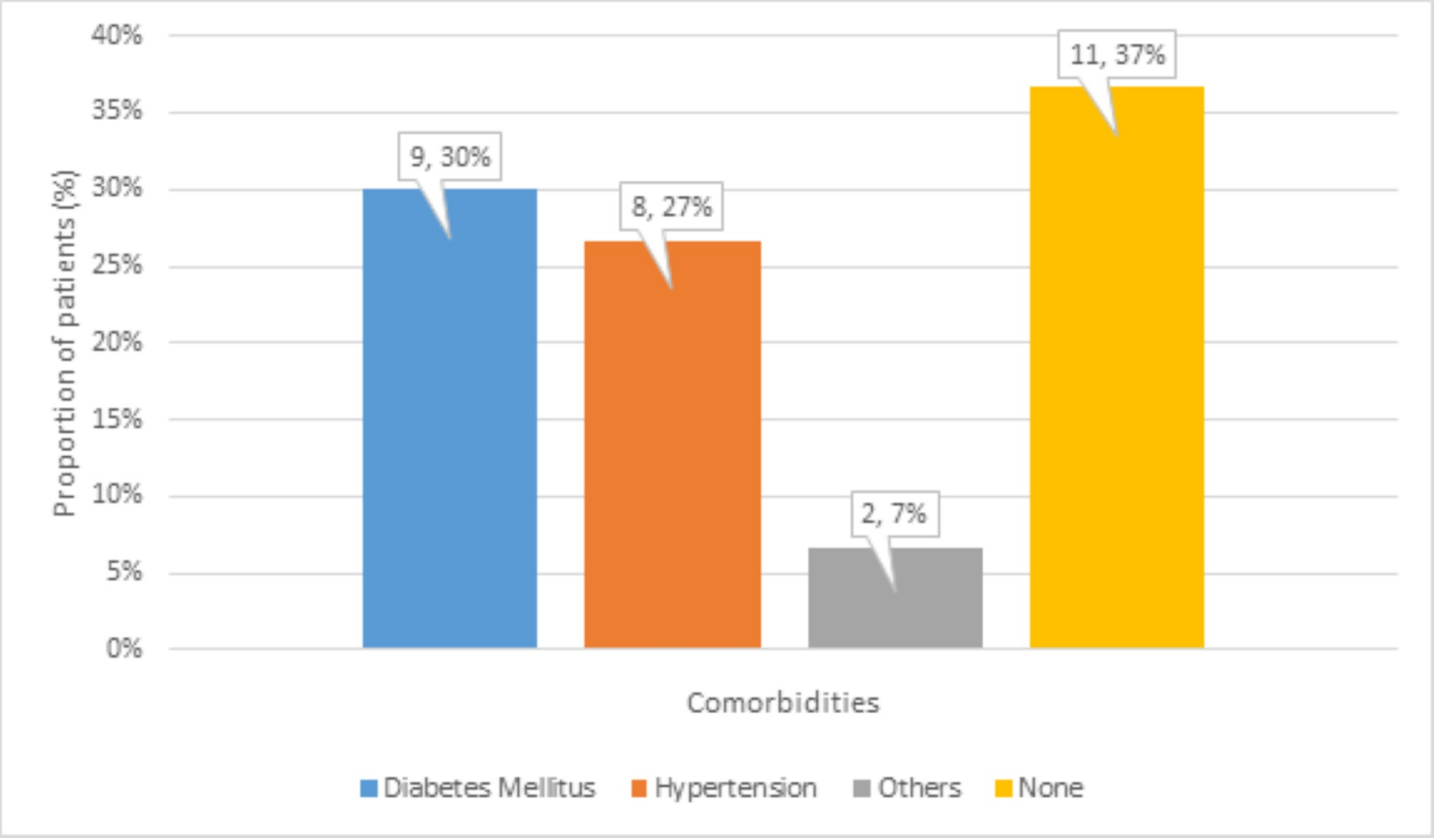
Distribution of comorbidities

There were 9 (30%) patients with diabetes mellitus, 8 (27%) patients with hypertension, 2 (7%) patients with other comorbidities, and 11 (37%) patients with no comorbidities (Figure 3).

**Figure 4:**
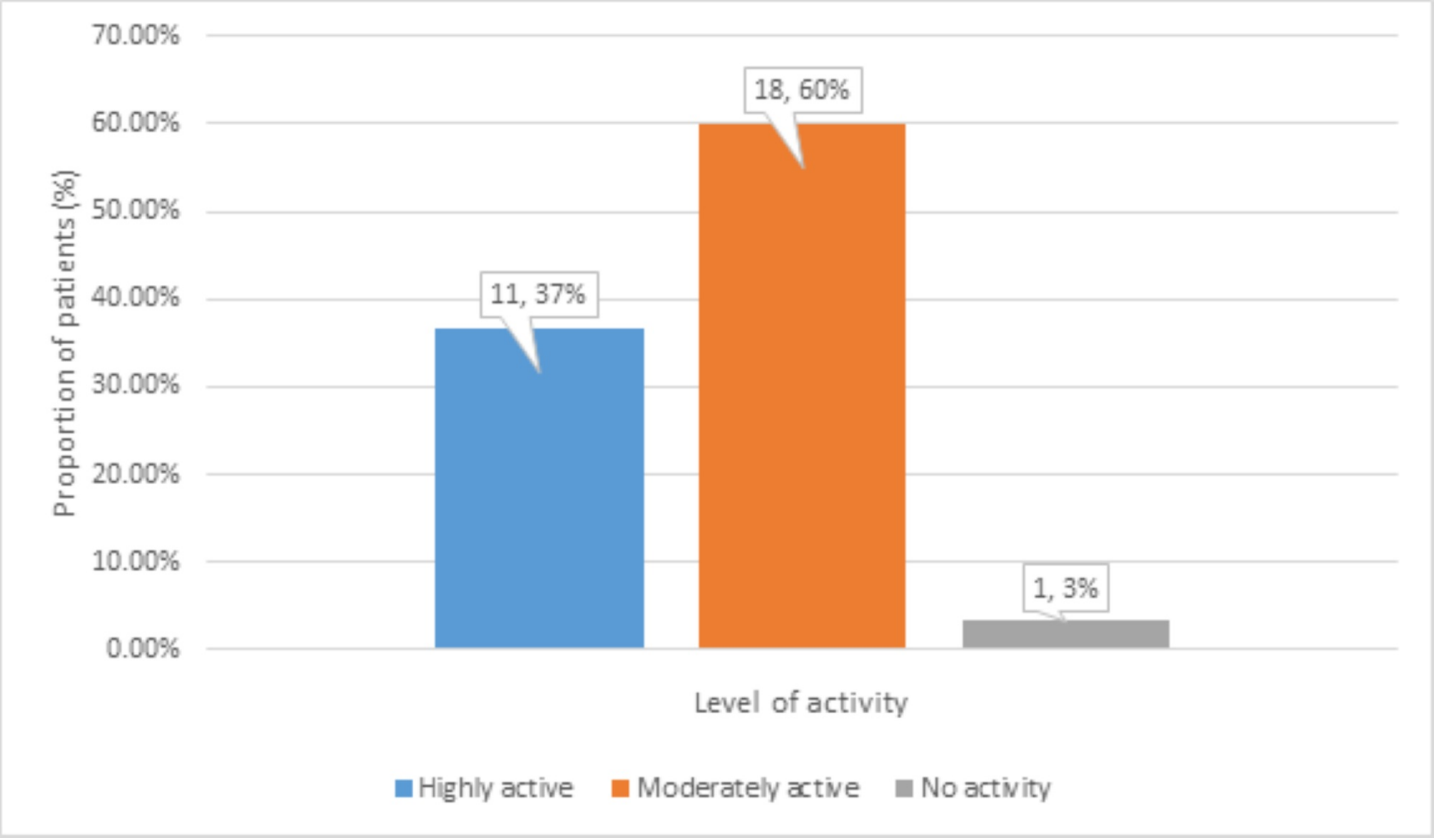
Distribution of level of physical activity before avascular necrosis

There were 11 (37%) highly active and 18 (60%) moderately active patients, and 1 (3%) non-active patient before developing avascular necrosis (Figure 4).

**Figure 5:**
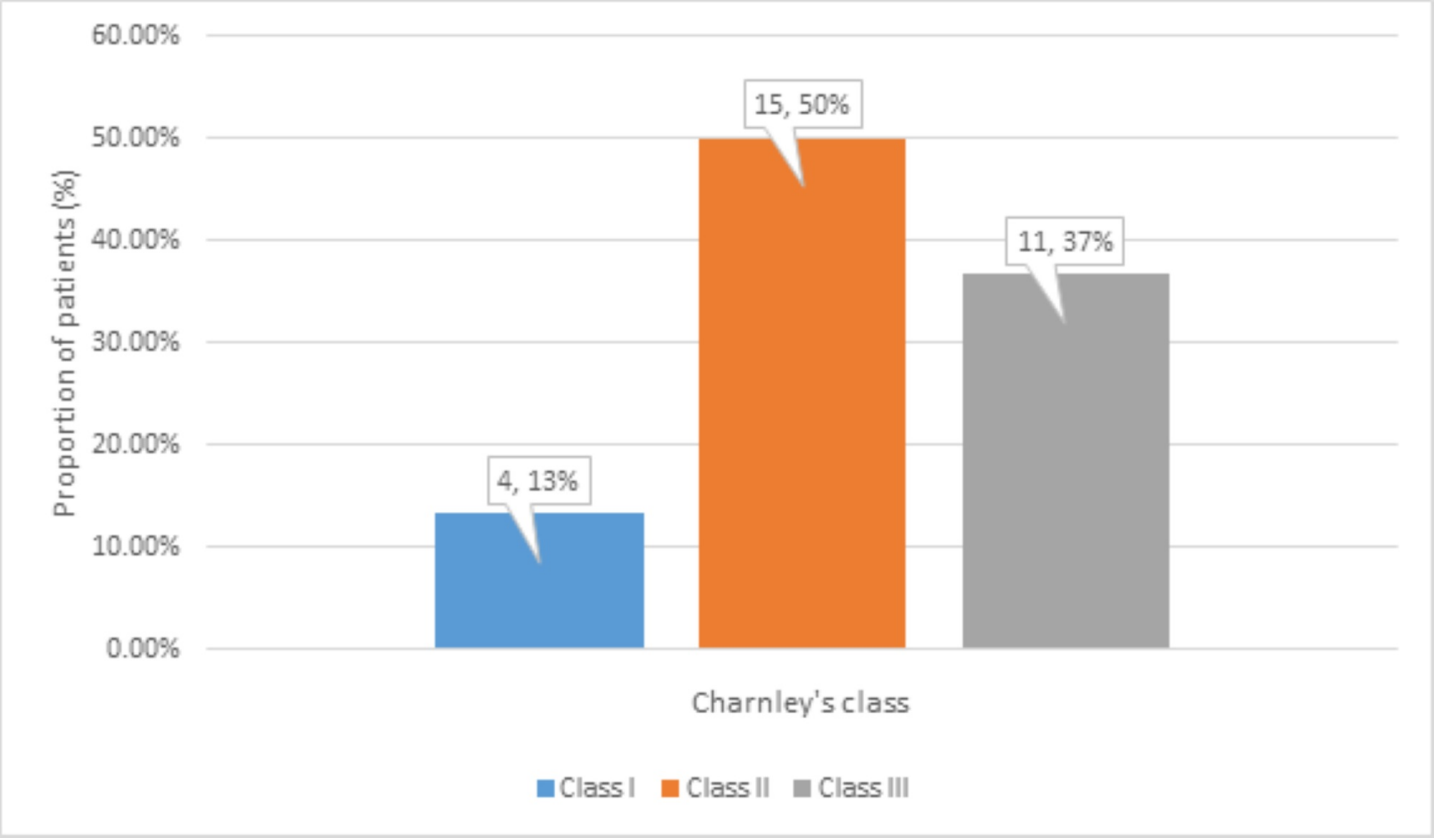
Distribution of Charnley’s classes

The effect of bilateral disease on total hip arthroplasty was determined by dividing patients into Charnley's classes. There were 4 (13%) patients in class I, 15 (50%) patients in class II, and 11 (37%) patients in class III (Figure 5).

**Figure 6:**
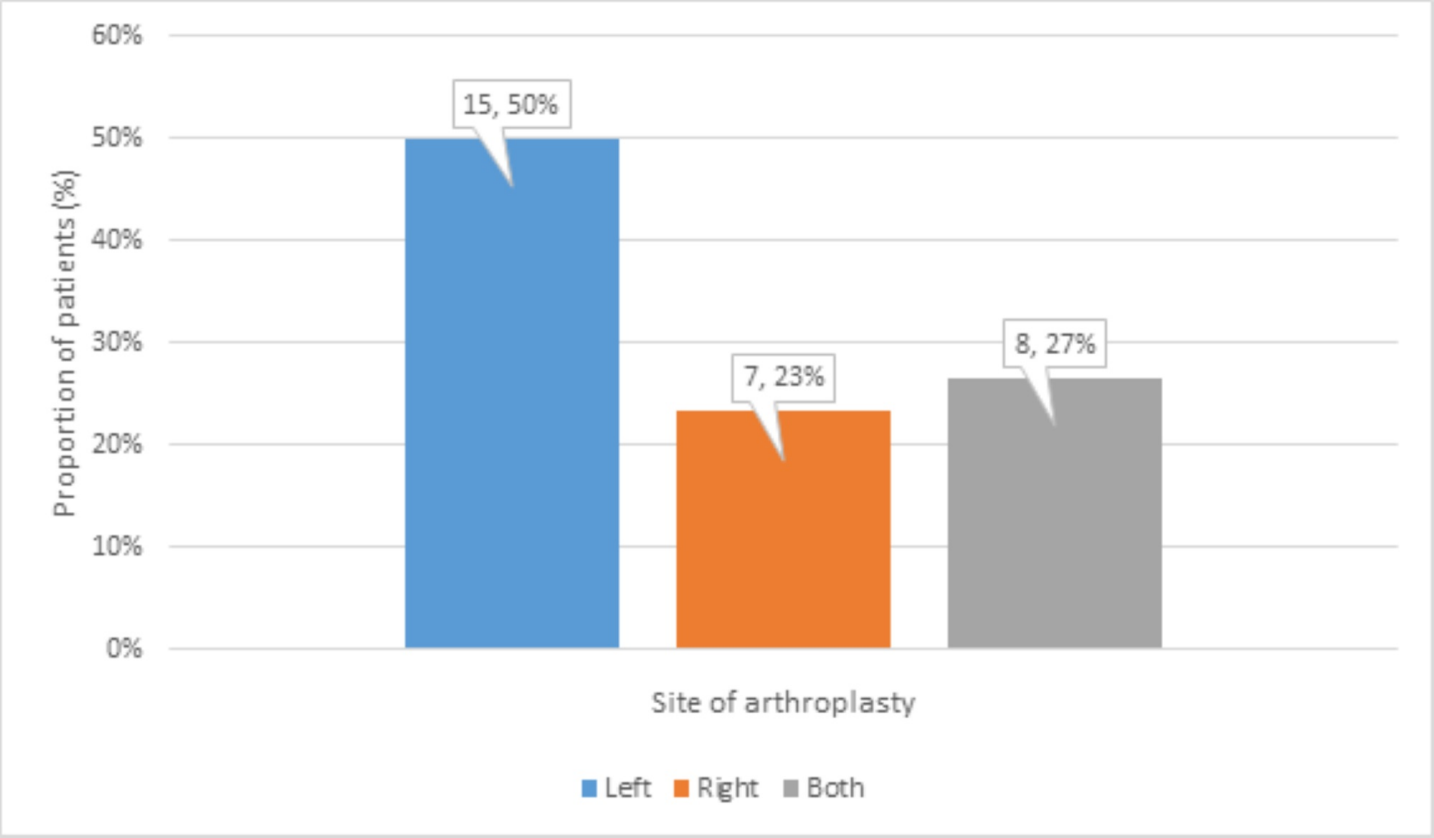
Distribution of site of arthroplasty

The arthroplasty was performed on the left side in 15 (50%) patients, on the right side in 7 (23%) patients, and bilaterally in 8 (27%) patients (Figure 6).

There were no significant early or late complications observed in this study. 

**Figure 7:**
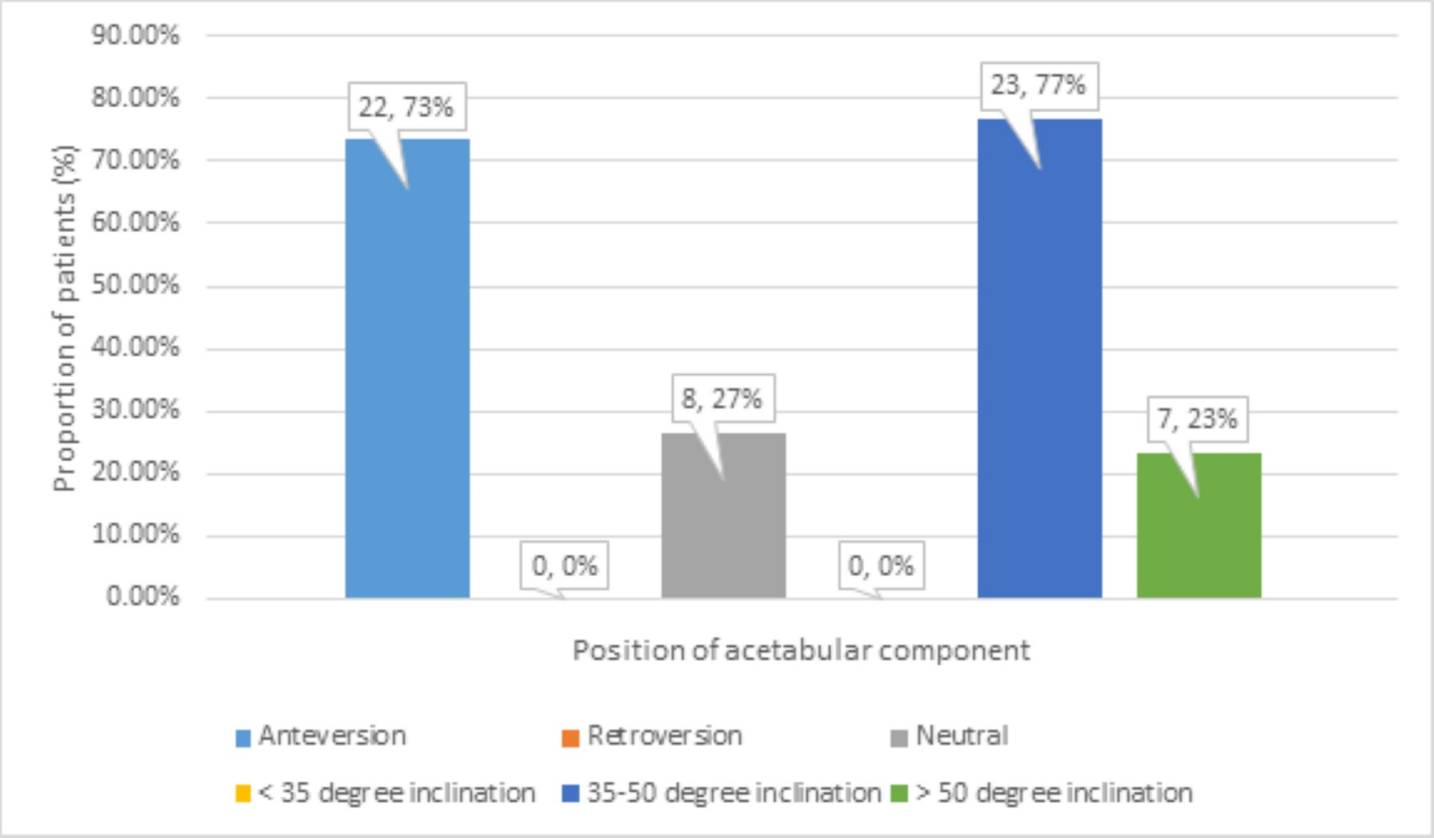
Distribution of position of acetabular component at follow-up

The position of the acetabular component on radiography was found to be anteverted in 22 (73%), retroverted in zero (0%), neutral in 8 (27%), <35^o^ inclined in zero (0%), 35^o^-50^o^ inclined in 23 (77%), and >50^o^ inclined in 7 (23%) patients at follow-up (Figure 7).

**Figure 8:**
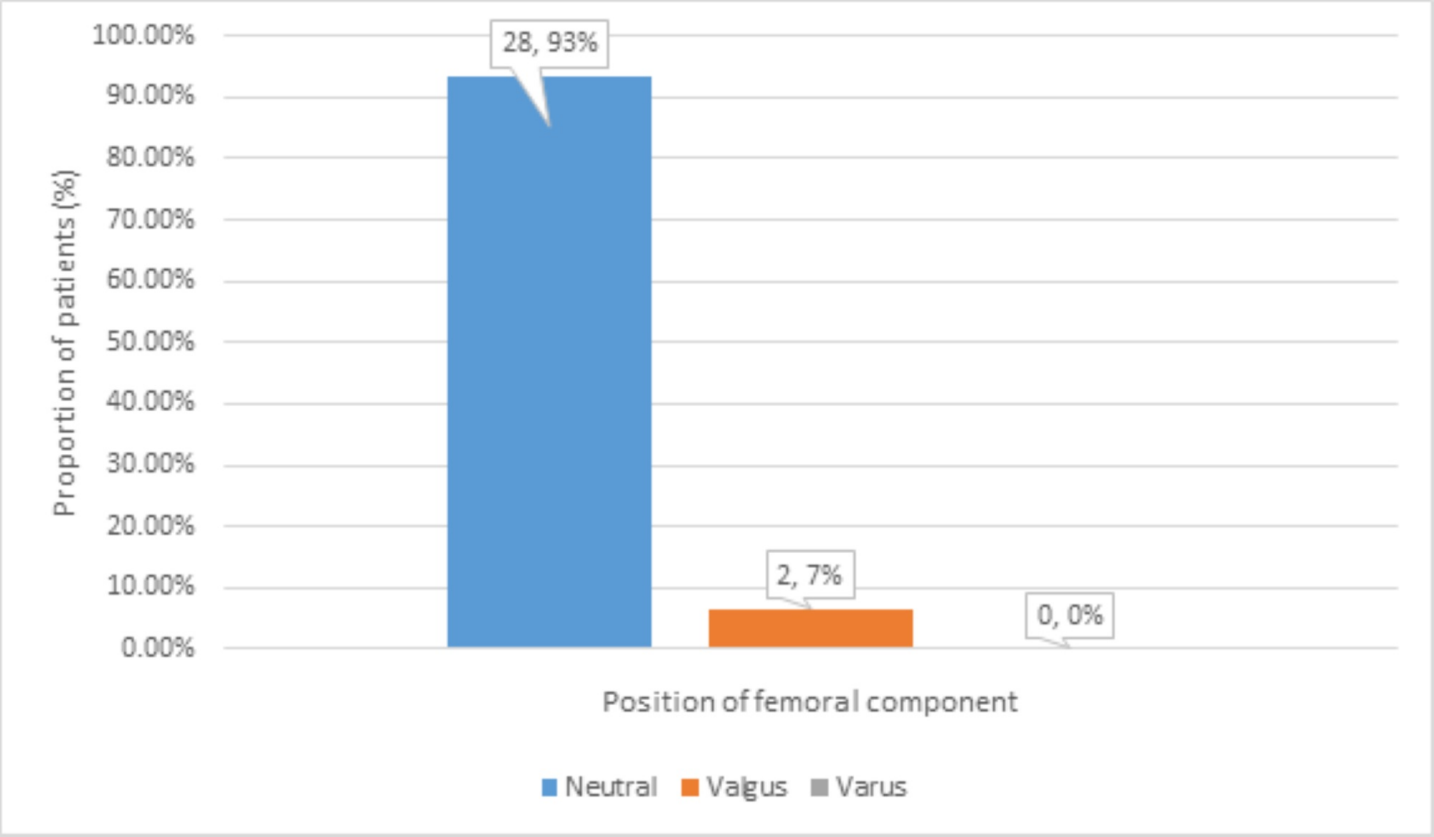
Distribution of position of femoral component at follow-up

The position of the femoral component on radiography was found neutral in 28 (93%), valgus in 2 (7%), and varus in zero (0%) patients at follow-up (Figure 8).

**Figure 9:**
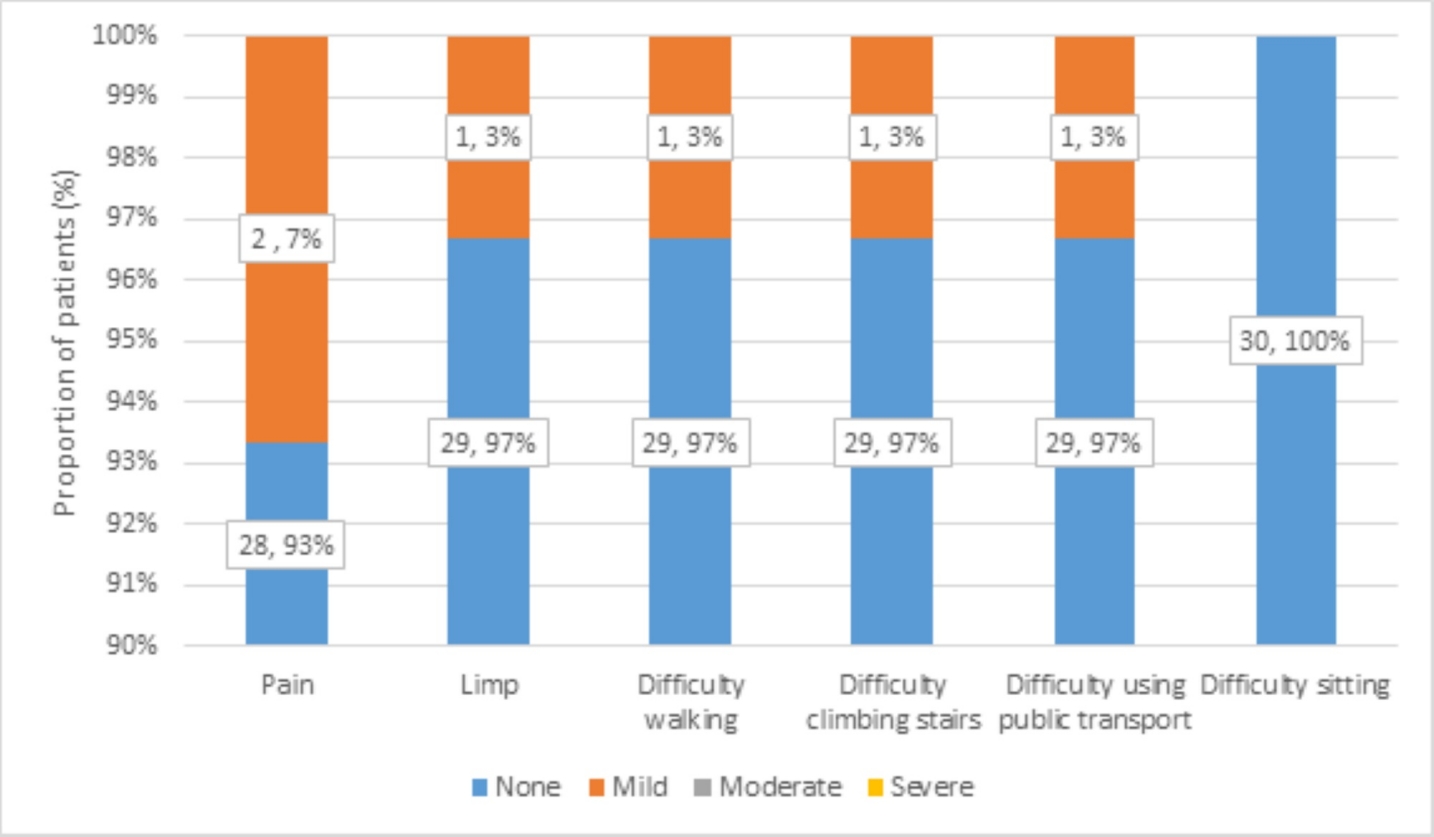
Distribution of functional assessment at follow-up

There was no pain in 28 patients (93%), while mild pain in 2 (7%) patients was observed. There was no limping, difficulty in walking, difficulty in climbing stairs, or difficulty using public transport in 29 (97%) patients, while mild limping, difficulty in walking, difficulty in climbing stairs, or difficulty using public transport in 1 (3%) patient was observed. There was no difficulty in sitting in 30 (100%) patients. This distribution of functional assessment at follow-up is shown in Figure 9.

All patients could easily wear shoes and socks, had no physical deformity, and attained normal limb length at follow-up. Only one patient needed a supportive device to walk.

There was no restriction in the range of movement postoperatively. This distribution of the restricted range of movement preoperatively and postoperatively is shown in Table 2.

**Table 2:** Distribution of restricted range of movement preoperatively and postoperatively

Restricted movement	Preoperatively	Postoperatively
Internal rotation	27 (90%)	None
External rotation	24 (80%)	None
Abduction	05 (17%)	None
Adduction	16 (53%)	None
Flexion	20 (67%)	None

 All 30 (100%) patients had 100^o^-110^o^ of flexion, 15^o^-20^o^ of abduction, 10^o^-15^o^ of external rotation, and 10^o^-15^o^ of adduction (Table 3).

**Table 3:** Distribution of degree of movement postoperatively

Degree	Flexion	Abduction	Adduction	External rotation
None	-	-	-	-
0^o^-8^o^	-	-	-	-
8^o^-16^o^	-	-	30 (100%)	30 (100%)
16^o^-24^o^	-	30 (100%)	-	-
24^o^-100^o^	-	-	-	-
100^o^-110^o^	30 (100%)	-	-	-

The overall performance was accessed at follow-up by using HHS and modified HHS. HHS was 100% in 27 (90%) patients, 96% in 2 (7%) patients, and 83% in 1 (3%) patient. The average HHS came out to be 99.2%. According to modified HHS, 29 (97%) patients had excellent and only 1 (3%) patient had a good outcome (Table 4).

**Table 4:** Modified Harris Hip Score (HHS)

Modified HHS	Patients
Excellent (90%-100%)	29 (97%)
Good (80%-90%)	1 (3%)
Fair (70%-80%)	-
Poor (0%-70%)	-
Total	30 (100%)

## Discussion

This study illustrates encouraging results in patients of avascular necrosis of hip who received cementless total hip arthroplasty over a minimum follow-up period of 12 months. Approximately 96.66% of patients were reported to perform all functions without any assistance, whereas only 3.33% of patients required assistance for walking. The HHS was documented at 100% in 90% patients, 96% in 6.66% patients, and 83% in only 3.33% of patients with an average HHS of 99.16%.

Avascular necrosis is a debilitating condition that often results in the collapse of the femoral head besides secondary osteoarthritis. Total hip arthroplasty is the recommended treatment for advanced stages of avascular necrosis and accounts for 5%-12% procedures conducted due to avascular necrosis [5,6].

The initially published literature about cemented total hip arthroplasty for avascular necrosis was disappointing. In 1988, Salvati et al. reported prognosis of cemented total hip arthroplasty in their patients with a failure rate of 37% over an average period of eight years. Furthermore, they reported a 100% failure in patients less than 30 years of age. Thus, they concluded that the probability of failure was four times greater in patients less than 30 years of age [7]. Another study, conducted between 1970 and 1984, reviewed the prognosis of 53 cemented total hip arthroplasties performed in 41 patients. A final follow-up was conducted after 10 years in 22 surviving hip arthroplasties that showed revision in eight hips, aseptic loosening in six hips, sepsis in one hip, and recurrent dislocation in one hip. Thus, they reported a poor prognosis of cemented total hip arthroplasty [8]. With the advent of new technology, outcomes of cemented total hip arthroplasty have improved over time. Kantor et al. reported a 96% success rate in 28 patients who underwent femoral reconstruction by second-generation cementing techniques with a mean follow-up for 7.7 years [9].

Another study published in 2014 compared the failure rate of cemented and uncemented total hip arthroplasty in patients older than 55 years of age. According to this study, patients with age 65 years or more depicted superior success rates with the use of cemented implants in comparison to uncemented implants, whereas the results noted for cemented and uncemented implants in patients with age 55-64 years were similar [10].

Uncemented total hip arthroplasty consists of the porous-coated prosthesis that encourages bone growth onto its surface. It was developed to provide an alternative to poor outcomes of early cemented total hip replacements. The initial results for cementless total hip replacements were poor as the smooth surface of cementless prosthesis prevented strong adherence to the bone causing aseptic loosening a few years’ post-surgery [11]. During the 1980s, the development of porous-coated material allowed bone ingrowth on the surface of prosthesis resulting in increased adhesion and improved prognosis [12]. Lins et al. published research in 1993 regarding the outcome of cementless total hip arthroplasty performed in the two years of 1984-1986 with a four- and six-year follow-up. The final results mentioned in her study showed an increase in average HHS from 47 to 86 points as well as a decrease in revision in patients who received cementless total hip arthroplasty [13]. Another encouraging study was published in 1994 in which Philips et al. reported prognosis of cementless total hip arthroplasty performed on 20 patients suffering from avascular necrosis reviewed over a minimum follow-up period of 24 months. They observed that the average HHS was 88 in 17 out of 20 hips [14]. Recently, a comparative study was conducted in 2017 that deduced the success rate of cemented and uncemented procedures for osteonecrosis. After two years, a final follow-up was conducted that showed reduced failure rates and less pain in patients who received the cementless procedure [15].

## Conclusions

Cementless total hip arthroplasty has reassuring results in patients <60 years of age and avascular necrosis of the hip with no other associated hip pathology. It is not associated with any significant early or late complications and has outstanding functional outcomes. Patients usually do not experience any pain, limping, difficulty in walking, difficulty in climbing stairs, difficulty using public transport, or difficulty in sitting. They can easily wear shoes and socks, have no physical deformity, and attain normal limb length. They develop no restriction in the range of movement. Therefore, patient satisfaction is high after cementless total hip arthroplasty for avascular necrosis of the hip. Further studies on a larger scale and with a longer duration of follow-up are needed to prove the validity of the above-stated outcomes.
